# TMK-VS-Pred: Impact of Decoy Choice on Target-Specific Scoring for Structure-Based Virtual Screening of Thymidylate Kinase, a Potential Anti-Mycobacterium Tuberculosis Target

**DOI:** 10.3390/molecules31213834

**Published:** 2026-10-23

**Authors:** Muhammad Junaid, Muhammad Zeeshan, Abbas Khan, Wenjin Li

**Affiliations:** 1 Institute for Advanced Study, Shenzhen University, Shenzhen 518060, China; juni_sjtu@sjtu.edu.cn; 2 School of Life Science and Biotechnology, Shanghai Jiao Tong University, 800 Dongchuan Rd., Minhang District, Shanghai 200240, China; 3 Department of Bioinformatics and Biotechnology, Islamic International University Islamabad, Islamabad 44000, Pakistan; 4 Department of Biomedical Sciences, School of Medical and Life Sciences, Sunway University, Sunway City 47500, Malaysia

**Keywords:** machine learning scoring, structure-based virtual screening, thymidylate kinase, TMK-VS-Pred

## Abstract

Artificial intelligence (AI) has revolutionized drug discovery by enhancing the efficiency and accuracy of structure-based virtual screening (SBVS) and binding affinity prediction. SBVS relies on scoring functions (SFs) to identify potential drug candidates, but conventional SFs often lack target specificity. AI-driven SFs overcome these limitations through data-driven approaches, improving predictive performance. Target-specific machine learning SFs (MLSFs) further enhance binding affinity predictions by capturing intricate molecular interactions, leading to more reliable virtual screening outcomes. This study focuses on thymidylate kinase (TMK) of Mycobacterium tuberculosis, a pivotal enzyme in nucleotide synthesis and DNA replication, making it a promising target for the development of new anti-TB therapies. Among the trained MLSFs, the ANN-PLEC classifier emerged as the top-performing model, achieving mean PR-AUC scores of 0.90. The corresponding F1 scores were 0.88. Our findings reveal that training and testing models on the same type of decoys introduces decoy bias, leading to overestimated SBVS performance. Importantly, ANN-PLEC demonstrated superior classification performance, achieving an NEF1% of 0.96. Additionally, XGB-GRID excelled in binding affinity prediction, with Pearson and Spearman correlation coefficients of 0.87 and 0.73, respectively, showcasing its capability to rank molecules accurately by binding affinity.

## 1. Introduction

Worldwide, tuberculosis (TB) affects millions of people annually [1]. Since Mycobacterium tuberculosis (MTB) grows slowly and has a strong tendency to develop resistance to commonly employed antibiotics, it is an especially challenging pathogen to battle [2]. There is a chance of life-threatening complications with this bacterial infection since it primarily targets the lungs [3]. In a report released by the World Health Organization (WHO), TB is the leading infectious cause of death, resulting in approximately 1.5 million deaths each year [4]. Even though TB is preventable and treatable, it remains a significant public health issue, particularly in developing nations [5]. In the 1950–1970s, the number of TB cases dropped dramatically as a result of the discovery of anti-mycobacterial agents such as rifampicin, ethambutol, pyrazinamide and isoniazid [6]. Due to multi-drug resistance in mycobacteria, there is currently a massive increase in TB cases throughout the world [7]. A growing number of drug-resistant TB strains, such as MDR-TB and XDR-TB, further complicate the fight against the disease [8,9]. To meet this challenge, novel TB drugs need to be developed, and measures need to be taken to limit its spread [10].

Several antibiotics are used in the initial treatment of TB, including rifampin (RIF), isoniazid (INH), pyrazinamide (PZA), and ethambutol (EMB) or streptomycin (SM), in order to prevent mutations that can occur if only one drug is used [11]. To eradicate some persistent bacilli, only rifampin and isoniazid are administered for four months [11]. TB is most effectively treated by INH and RIF. In combination with these two drugs, PZA has a high sterilization effect and seems to act on the semidorment MTB, which is not affected by other TB drugs. It is similar to nicotinamide in that PZA is not soluble in water, and it is converted within the cell into its active form by the PZase enzyme.

DNA, the genetic blueprint of life, is synthesized through nucleotide synthesis, in which thymidylate kinase (TMK) plays a central role. The TMK enzyme contributes to maintaining the integrity of genetic material by being an important component of deoxythymidine monophosphate synthesis and responsible for the conversion of thymidine monophosphate (dTMP) into thymidine diphosphate (dTDP), leveraging adenosine triphosphate (ATP) as the source of the phosphoryl group [12]. This enzyme belongs to the family of enzymes known as kinases, which transfer phosphate groups from high-energy donor molecules to specific targets [13]. It has been reported that TMK is an attractive target for the development of novel anti-TB drugs in the context of MTB [12,14]. TMK inhibition eventually leads to bacterial death as it impairs the production of DNA replication-related deoxythymidine triphosphate (dTTP). In the fight against TB, the dTTP synthesis pathway plays a critical role due to its strategic location. There have also been reports of thymine non-nucleoside derivatives that are potent MTB-TMK inhibitors, although most of them are thymidine monophosphate analogs [15,16]. The inhibitory effects of some non-nucleoside derivatives on this enzyme have also been reported [17,18].

The first crystal structure of TMK-MTB was solved at a resolution of 1.95 Å with the natural substrate TMP [19]. The three-dimensional crystal structure of MTB-TMK shows similar folding to other bacterial TMKs. TMK-MTB comprised 214 amino acids with 24 kDa. The important residues that are required for proper functioning of this enzyme are situated in three regions, namely P-loop, LID and the loop containing the strictly conserved arginine residue. In the first place, there is the P-loop motif, which has the consensus sequence GxxxxGKS/T. An important function of this motif is to guide the precise positioning of phosphoryl groups from the phosphate donor. Second, there is a conserved arginine residue in the loop that brings the acceptor and donor nucleotides together. The consensus sequence is DR(Y/H/F). The final region is the LID region, which features a flexible segment that undergoes closure when phosphorylated.

The SBVS approach leverages the 3D structure of the target protein and has proven very useful in the drug discovery process [20,21,22,23]. In SBVS, computational tools screen a huge library of small molecules for a subset of potent molecules likely to be potent binders of the target protein. Molecular docking is a computational tool commonly used in the SBVS tasks [24]. The availability of experimental protein structures and binding affinity data for their cognate ligands supports docking to predict protein–ligand binding affinity and binding mode. Docking is considered the backbone of SBVS, which entails two major challenges: 1) the correct pose of the molecule must be sampled; 2) selection and ranking of sampled poses. Many methods exist for the former task. The latter step serves to guide the sampling process and rank the sampled poses. Putative pose scores can direct the sampling strategy, as seen in Vina, or evaluate the fitness of an entire population, as in AutoDock4 [25,26].

An accurate SF is crucial for ranking and choosing samples in any circumstance [27]. It is necessary to develop approaches that consider the nonlinear relationships between the protein–ligand characteristics and the ligand’s binding affinity and bioactivity, as these linkages should not be ignored [27,28]. Docking algorithms driven by machine learning (ML) have found molecules for a range of targets and account for nonlinearities. An SF is used to score docked molecules based on their estimated affinity/energy of interactions with the target. The post-screening hit list should prioritize tightly bound molecules according to the accuracy of their SF predictions [23]. Classical SFs depend on linear assumptions and provide a hit list, which is sometimes rather restricted yet beneficial in certain instances [29]. To rescore docked poses, a target-specific MLSF makes good use of the data that is already available for the examined target.

Both generic and target-specific MLSFs have had a profound impact in the field of drug discovery, owing to their power being utilized for pose prediction tasks [30]. These state-of-the-art MLSFs process large datasets to learn patterns that most classical tools overlook. However, MLSFs’ performance is highly sensitive to the type and quality of the training data. MLSF training datasets have two main classes of molecules: actives and inactives. Active molecules bind to the target protein with high affinity, whereas inactives either do not bind at all or bind with much lower affinity. Selecting high-quality inactives is essential since it influences the model’s capacity to differentiate between real binders and non-binders [22]. Inactives fall into three broad categories: true inactives: These are molecules that have shown no evidence of binding to the target protein in experimental validation. Incorporating true inactives into the training dataset provides the MLSF with realistic examples of non-binders, which improves its performance in making predictions. However, in many situations, the quantity of true inactives is insufficient for MLSFs to learn patterns effectively. DeepCoys: These non-binders are generated from active molecules and closely resemble the physicochemical characteristics of active molecules but have different structures [31]. They pose a challenge to the MLSF by providing examples that are hard to distinguish, which can make the model more resilient. Random decoys: These are randomly chosen from databases such as ZINC and considered non-binders. Random decoys have different physicochemical and structural properties. Although more accessible, Random decoys might not always pose a practical challenge to the MLSF, potentially leading to overestimated performance. The choice of decoys used for training is a critical factor affecting an MLSF’s prediction performance. Decoys that are too different from actives can result in an MLSF that performs well on training data but poorly on new, unseen data. Conversely, decoys that are overly similar to actives can make training more challenging but can ultimately result in a more robust and reliable SF.

The most widely used benchmark resource for decoy-based evaluation of molecular docking and virtual screening is the Directory of Useful Decoys, Enhanced (DUD-E), which succeeded the original DUD benchmarking sets [32]. For each of its 102 targets, DUD-E pairs experimentally annotated actives with 50 property-matched decoys drawn from ZINC. Candidate decoys are chosen to match each active across six one-dimensional physicochemical descriptors—molecular weight, calculated logP, number of rotatable bonds, hydrogen-bond donors, hydrogen-bond acceptors and net charge—while remaining topologically dissimilar to it in terms of Morgan-fingerprint Tanimoto similarity. The intent of this matching is that enrichment cannot be obtained simply by separating actives from decoys on bulk physicochemical properties, so that reported enrichment reflects recognition of the binding mode rather than a trivial property gradient. DUD-E has since become the de facto standard both for reporting virtual screening enrichment and for training generic machine learning scoring functions. Subsequent analyses have nevertheless shown that DUD-E-style sets retain exploitable biases, including analog bias among the actives and residual artificial enrichment in the decoys, which permit models to learn dataset-specific signatures instead of genuine protein–ligand recognition and consequently to report performance that does not transfer to prospective screening.

Three considerations motivated the use of DeepCoy and randomly selected ZINC20 decoys in the present work rather than established DUD-E decoys. First, thymidylate kinase of M. tuberculosis is not among the 102 DUD-E targets, so no curated DUD-E decoy set exists for TMK-MTB; a DUD-E-like set would in any case have to be generated de novo by applying the DUD-E property-matching protocol to our own curated actives. Second, DeepCoy may be regarded as a methodological successor to that protocol: rather than mining a library for compounds that happen to match an active on a handful of descriptors, it uses a graph-generative neural network to construct property-matched decoys de novo, and it was reported to yield lower DOE scores (indicating better embedding of the actives in property space) and lower doppelganger scores (indicating a smaller risk of latent actives) than DUD-E decoys generated for the same active molecules [32]. DeepCoy therefore supplies, for an arbitrary target, a property-matched decoy set at least as demanding as a DUD-E set. Third, the central question addressed here is how decoy difficulty shapes both the apparent and the transferable performance of a target-specific scoring function, which requires two decoy sets at opposite ends of the difficulty spectrum; DeepCoy decoys and Random ZINC20 decoys provide exactly that contrast, with DUD-E decoys occupying an intermediate position between them. Benchmarking TMK-VS-Pred against decoys built with the DUD-E protocol, and against DUD-E targets themselves, remains a worthwhile extension of this study.

In this manuscript, we have conducted a thorough analysis to demonstrate that models trained with DeepCoys exhibit greater robustness. Several important elements contribute to the phenomenal performance of DeepCoy-trained models. Firstly, the increased complexity of DeepCoys provides a more diverse and harder set of molecules, which causes the model to acquire subtler and generalizable properties. This increases the likelihood that the model will be successful. The second benefit of using DeepCoys models is that they are meant to imitate the characteristics of true positives more accurately. This helps to reduce the possibility of biases being introduced into the training data, which are often caused by Random decoys. The resemblance between DeepCoys and true positive instances necessitates the model to build more refined discrimination skills, which leads to improved discrimination as a result of this decreased bias. Furthermore, by training on these more challenging cases, the model enhances its generalization skills, which in turn makes it better suited to handle a broader variety of situations in applications that are used in the real world. Last but not least, the increasing complexity of the training challenge leads to a model that demonstrates a greater level of intelligence. This is because the model is required to create more complex methods in order to differentiate between DeepCoys and active molecules. In summary, this manuscript aims to showcase the superior robustness of models trained with DeepCoys, highlighting their enhanced generalization capabilities and refined discrimination skills, ultimately contributing to the development of more reliable and effective therapeutic strategies against tuberculosis.

## 2. Material and Methods

### 2.1. Preparation of Actives

Three databases were curated for biochemical assays against TMK-MTB: BindingDB, PubChem and ChEMBL [33,34,35,36]. These databases stored experimental activity data of molecules against their respective targets in the form of IC_50_, K_i_ and K_d_. Data for 307 molecules were collected from BindingDB, for 360 from PubChem and for 360 from ChEMBL. Molecules were discarded if their IC_50_ or K_i_ values were missing. In the present study, for the sake of convenience, we treated IC_50_ and Ki and kept both of them. After duplicate removal based on SMILES notation and similar molecule removal among the three datasets, the final dataset contains 674 molecules.

### 2.2. Preparation of Inactive Molecules

Based on PubChem’s cutoff criteria, where molecules exhibiting activity below 10 µM are classified as active and those above are deemed inactive, the dataset of 674 molecules was divided into 286 active and 388 inactive compounds. In natural scenarios, the proportion of active molecules is exceedingly low compared to inactive ones. To better reflect this natural imbalance, additional inactive molecules were generated using the DeepCoy algorithm [31]. DeepCoy utilizes graph-generative neural networks to construct property-matched decoys. The SMILES of the active molecule are used as input for the DeepCoy algorithm, and it generates structurally dissimilar molecules with the same physicochemical properties [31]. A hundred decoys per active molecule were generated, and finally fifty decoys per active molecule were kept based on DOE score and doppelganger score [31]. The selection of fifty decoys per active molecule is based on established protocols and ensures the generation of a structurally diverse set of decoys [20,32,37]. To determine how well an active is embedded in chemical space, DOE scores use receiver operating characteristic (ROC) curves calculated using its physicochemical properties. DOE score is calculated as the average absolute difference between these ROC curves and a random distribution. A DOE score of zero is obtained by optimal embedding of actives and decoys, while a DOE score of 0.5 is obtained by complete separation of the actives and decoys in physicochemical space. An evaluation of the structural similarity between active compounds and the most closely related decoys is assessed by the doppelganger score.

The terminology and the experimental status of the inactive molecules used here warrant explicit clarification. Only the 388 compounds derived from the curated bioactivity data are experimentally characterized non-binders: these molecules were assayed against TMK-MTB and reported activities weaker than the 10 µM PubChem cutoff, and they are referred to throughout as true, or experimentally confirmed, inactives. The DeepCoy decoys and the Random ZINC20 decoys, by contrast, have never been tested against TMK-MTB. They are presumed non-binders, assigned that label by the standard benchmarking convention that a property-matched or randomly drawn library compound is overwhelmingly unlikely to be active against any given target. This is an assumption of the benchmark rather than an experimental measurement, and it does not establish true biological inactivity. Accordingly, wherever the words “inactive” and “decoy” are used in the remainder of this manuscript, “decoy” always denotes a computationally assigned presumed non-binder, while “true inactive” denotes an experimentally confirmed one; the implications of this distinction for the interpretation of our performance metrics are discussed in the Limitations and Future Directions Section.

### 2.3. Molecular Docking

To generate protein–ligand complexes, molecular docking was performed utilizing smina [38]. Smina is a fork of AutoDock Vina that enhances the minimization and scoring [25]. It is available under a GPL2 license at http://smina.sf.net (accessed on 19 October 2026). The crystal structure with bound substrate TMP was used as a receptor for docking (PDB ID 1GTV). The ligand search space was defined using the coordinates of the existing substrate. The exhaustiveness was set to 8. The binding mode was set to 1 to generate one dock pose for each ligand. The inbuilt smina scoring was used to score and rank the docked poses. Smina used both gradient and Monte Carlo steps to search and rank poses. The docked poses were saved in SDF format.

### 2.4. Vector Representation of Protein–Ligand Complexes

In the present study, the protein–ligand extended-connectivity (PLEC) fingerprint was calculated to represent the protein–ligand interactions of the complex. PLEC fingerprints encode the extended-connectivity fingerprint environment of the protein–ligand atoms that are at specific distances from each other [39]. As originally presented in the ECFP, Rogers and Hahn’s concept of atom environments has been developed for the PLEC fingerprint [40]. Four main steps are involved in calculating PLEC fingerprints. The first step in the generation of PLEC fingerprints entails the identification of atoms of ligand and protein that interact with each other at a specific cutoff distance. The second step is the identification of neighboring atoms and bond connectivity up to a certain depth. The third step is the assignment of an identifier to each atom. The fourth step is the derivation of the position of ‘1’ bits in the fixed-length binary vector representation. The values of the four major parameters—cutoff distance, protein depth, ligand depth, and vector length—were set to 4.5 Å, 4, 2, and 1092, respectively. Open Drug Discovery Toolkit was used to calculate the PLEC fingerprints [41].

The process of GRID featurization in this study involves several key steps using the RDKitGridFeaturizer from the DeepChem Python package. Initially, a box centered around the centroid of the docked ligand encapsulates each protein–ligand complex. The dimensions of this box, determined by the ‘box_width’ parameter, were set to a default of 16 Å. Inside this box, a grid of uniformly sized voxels is established, with each voxel also measuring 16 Å across, in line with prior research recommendations [42,43]. Within each voxel, various types of interaction fingerprints are generated to capture the dynamics within the protein–ligand complex. These include intra-ligand, intra-protein, and protein–ligand interactions, focusing on atoms that are within 4.5 Å of any ligand atom. The intra-ligand and intra-protein fingerprints utilize ECFPs, with a set radius ‘r’ and 2 n bits resulting in a 512-bit vector, where ‘ecfp_degree’ was set to 2 and ‘ecfp_power’ to 9. Protein–ligand interactions within the voxel are further refined by categorizing intermolecular distances into three distinct bins: 0–2 Å, 2–3 Å, and 3–4.5 Å. For each distance range, Structural Protein–Ligand Interaction Fingerprints (SPLIFs) are computed, employing 2 m bits (‘splif_power’ was set to 9) and hashed into a 512-bit vector per bin, accumulating to 1536 bits across all bins. Additionally, this featurization process tracks the number of hydrogen bonds and salt bridges within specified distances, enhancing the granularity of the interaction data. These interactions are recorded within distance ranges of 2.2–2.5 Å, 2.5–3.2 Å, and 3.2–4.0 Å for each voxel. The compilation of all intra-voxel features results in a comprehensive 2052-bit fingerprint for each voxel, providing a detailed characterization of the interactions within the protein–ligand complex.

### 2.5. Model Training

Three machine learning models—Random Forest (RF), XGBoost (XGB), and ANN—were trained on PLEC fingerprints. The hyperparameters of each model were tuned using a Bayesian optimization algorithm using hyperopt [44]. In Random Forest, multiple decision trees are combined to improve prediction accuracy [45]. Using a bagging technique creates diverse trees and reduces overfitting, making it suitable for a wide range of data types and robust against outliers. The XGBoost algorithm, on the other hand, is an extreme gradient-boosting algorithm [46]. A sequence of decision trees is built sequentially, with errors corrected in each iteration, using gradient boosting and regularization techniques for high accuracy. It is suitable for both regression and classification tasks, and XGBoost is known for its speed and efficiency. ANN was used to be inspired by the human brain. ANN consists of an input layer, one or more hidden layers, and an output layer. Each hidden layer is composed of multiple neurons. In most virtual screening tasks, these algorithms have been used in their regression or classifier variants in combination with PLEC and GRID fingerprints, offering high-performing models that were proven better than most classical SFs [20,39,47]. In the present study, we would like to determine whether these combinations of features along with more challenging datasets would still be able to perform well.

### 2.6. Evaluating ML Models

We trained both the classification and regression variants of each algorithm. We would like to determine whether classification or regression variants will be suitable for the classification task. For classification, an optimized threshold was determined based on the F1 score. This threshold was then used as a cutoff: molecules with probability scores above the threshold were classified as active, while those with scores below were classified as inactive. For regression variants, we predicted the binding affinity score in terms of pIC50. So, the output predicted value for each molecule is a continuous value. Previously, we assigned a low pIC50 value of 2 to all decoys [20]. Based on PubChem’s criteria, a pIC50 value of 5 (corresponding to 10 µM) was used as the threshold: molecules with predicted pIC50 values equal to or greater than 5 are classified as active, while those with lower values are considered inactive. In this way, the continuous predicted values of regression models were converted to active and inactive. For the classification task, four metrics were utilized—PR-AUC, Precision, Recall, and F1—while for regression models, the MAE was used along with the four classification metrics. We preferred PR-AUC instead of ROC-AUC because the dataset was highly imbalanced (50 decoys per active). The choice was made for the following reasons:

- There is usually a greater significance placed on the minority class in imbalanced datasets (actives). PR-AUC measures how well a model identifies the minority class instances, taking into account Precision (the percent of true positives among all positive predictions) and Recall (the percent of true positives identified among all actual positives.
- ROC-AUC depends on the number of inactive or decoys, as it considers both the true positive rate TPR and the False positive rate FPR. In an imbalanced dataset, there is a huge number of decoys, and the model learns more for decoys, resulting in more predicted negative instances and a high ROC-AUC value.
- PR-AUC is more sensitive to imbalanced datasets as it does not consider the decoys. It focuses only on how many active molecules are correctly classified while minimizing false positives. This makes PR-AUC more informative when evaluating models on datasets with a large number of decoys per active.

During 5-fold CV and testing, the PR-AUC, Precision, Recall, F1, and MAE were used and imported from the ‘metrics’ module of the ‘sklearn’ Python library (v.1.0.2): ‘roc_auc_score’, ‘precision_score, ‘auc’, ‘matthews_corrcoef’, ‘recall_score’, ‘precision_recall_curve ’ and ‘mean_absolute_error’.

We also calculated the ranking power in terms of the enrichment factor. The enrichment factor can be calculated at specific percentages such as 1, 3, etc., and hence is called EF1%, EF3%, and so on. EF1% can be calculated as the fraction of actives in the top X% divided by the fraction in the whole dataset. EF1% can be calculated with the following formula:

EF1%=The number of actives at top 1%the number of total molecules at top 1%the total number of activesthe total number of molecules

### 2.7. Data Partitioning and Prevention of Structural Data Leakage

**Figure 1:**
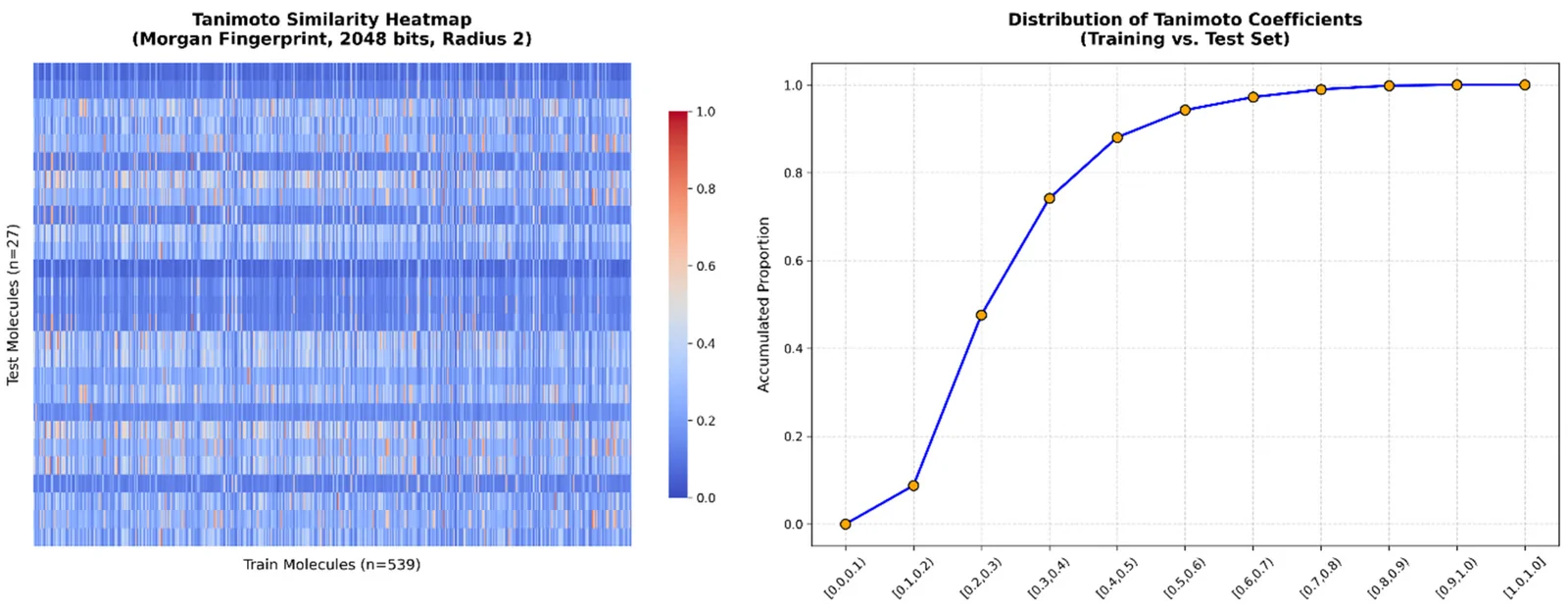
Molecular similarity analysis between training and test sets. (**Left**) Tanimoto similarity heat map of test molecules (n = 27) against training molecules (n = 539) using Morgan fingerprints (2048 bits, radius 2). Each cell represents the Tanimoto coefficient between a test and training molecule, with colors indicating similarity levels (blue: low similarity, red: high similarity). (**Right**) Cumulative distribution of Tanimoto coefficients, showing the proportion of test molecules with at least one training molecule above a given similarity threshold. The majority of test molecules exhibit low to moderate similarity to training molecules, indicating a diverse test set.

The 674 curated molecules were not partitioned at random. Splitting was performed with a chemical-similarity-constrained clustering strategy, implemented with the Butina clustering splitter (ButinaSplitter) of the DeepChem package applied to Morgan fingerprints with a Tanimoto distance metric. Butina clustering first groups structurally related molecules into clusters, after which whole clusters, rather than individual molecules, are assigned to the training or the test partition. Because every member of a cluster is kept together, no close structural analog of a test molecule is left behind in the training set, and this is the mechanism by which structural data leakage between the two partitions is prevented. This clustering-based strategy serves the same purpose as a Bemis–Murcko scaffold split and is in some respects the stricter of the two, because clusters are defined by whole-molecule fingerprint similarity rather than by the identity of the ring scaffold alone: two molecules that share a Bemis–Murcko scaffold but differ substantially in their substituents may be placed in opposite partitions by a scaffold split, yet are grouped together by Butina clustering. The resulting partitions were verified post hoc, and the Tanimoto similarity between training and test actives was confirmed to remain below 0.8 (Figure 1). We did not additionally perform an explicit Bemis–Murcko scaffold split; because the two schemes control chemotype overlap by different criteria, reporting both would be informative, and a scaffold-split replicate is accordingly identified as a priority in the Limitations and Future Directions Section.

### 2.8. Statistical Analysis

All statistical comparisons between modeling choices were performed with the two-sided independent two-sample Student’s *t*-test, as implemented in the SciPy Python library (scipy.stats.ttest_ind), retaining the default assumption of equal population variances. For each comparison the two groups consisted of the performance values (PR-AUC or F1) obtained for the two modeling configurations being contrasted—PLEC versus GRID features, DeepCoy versus Random decoys, classification versus regression variants, and target-specific machine learning scoring functions versus generic scoring functions—pooled across the corresponding training–test runs. Statistical significance is reported throughout at the thresholds *p* < 0.001 (highly significant), *p* < 0.01 (more significant) and *p* < 0.05 (significant), with *p* ≥ 0.05 regarded as not significant. Every *p*-value quoted in the text and in the figures was obtained with this test, and no correction for multiple comparisons was applied. We note that, because the configurations being compared are evaluated on the same molecules, the two groups in each comparison are not strictly independent, so that a paired alternative such as the paired *t*-test or the Wilcoxon signed-rank test would also be defensible. The unpaired test used here is generally the more conservative of the two, since it does not exploit the pairing to reduce the variance of the difference, and the comparisons that we report as significant would therefore not be weakened by adopting a paired test.

## 3. Results and Discussion

### 3.1. Experimental Design

The experiment starts with retrieving the target structure. The keyword “thymidylate kinase” was searched in the PDB databank; initially, the search was limited to crystal resolution ≤1.5 Å. Since no structures met this criterion, the search was expanded to include resolutions between 1.5 Å and 2.5 Å. Nineteen crystal structures were found, and the one with the highest resolution of 1.55 Å (PDB ID 1GTV) was selected for further experimentation. 674 molecules were curated from the PubChem, ChEMBL, and BindingDB databases. All the IC50 values were converted to pIC50 using our in-house Python code. The minimum and maximum pIC50 are 5 and 9.7, respectively, for actives and less than 5 for inactives, indicating that our dataset contains highly potent molecules within the nM and µM range.

This was followed by the generation of the decoys. One hundred decoys were generated, and 50 optimized decoys per active molecule were used further and named DeepCoy decoys. The selection of 50 decoys per active molecule is based on established protocols and ensures the generation of a structurally diverse set of decoys [20,32,37]. As we aimed to analyze the performance of ML models trained with challenging decoys such as DeepCoy decoys, we also included Random decoys. Another reason for choosing these decoys is that the training and testing sets should not have decoys generated through the same methods to reduce bias. As a result, two training datasets were designed:

1. The first training dataset was designed to have 80% of true active and true inactive molecules form the total molecules and their corresponding DeepCoy decoys with a ratio of 1:50.
2. The second training dataset was designed to have 80% of true active and true inactive molecules form the total molecules and their corresponding Random decoys with a ratio of 1:50. The Random decoys were selected randomly from the ZINC20 database [48].

Two types of test sets were designed:

1. The first test set was designed to have 20% true active and inactive molecules with their corresponding DeepCoy decoys.
2. The second test set was designed to have 20% true active and inactive molecules with Random decoys.

The Tanimoto similarity between our training and test set active molecules was ensured to be less than 80% using the Morgan fingerprint (Figure 1).

It was expected that Random decoys would not be challenging, and ML models could perform better regardless of the training dataset. The test set with DeepCoy decoys offers challenging tasks, and ML models may struggle to perform, especially those trained with Random decoys. In summary, eight different scenarios were designed (Table 1). We also expect that ML models trained with either DeepCoys or Random decoys will perform better on the test having the decoys of the same origin.

### 3.2. Evaluating Model Robustness with Cross-Validation

**Figure 2:**
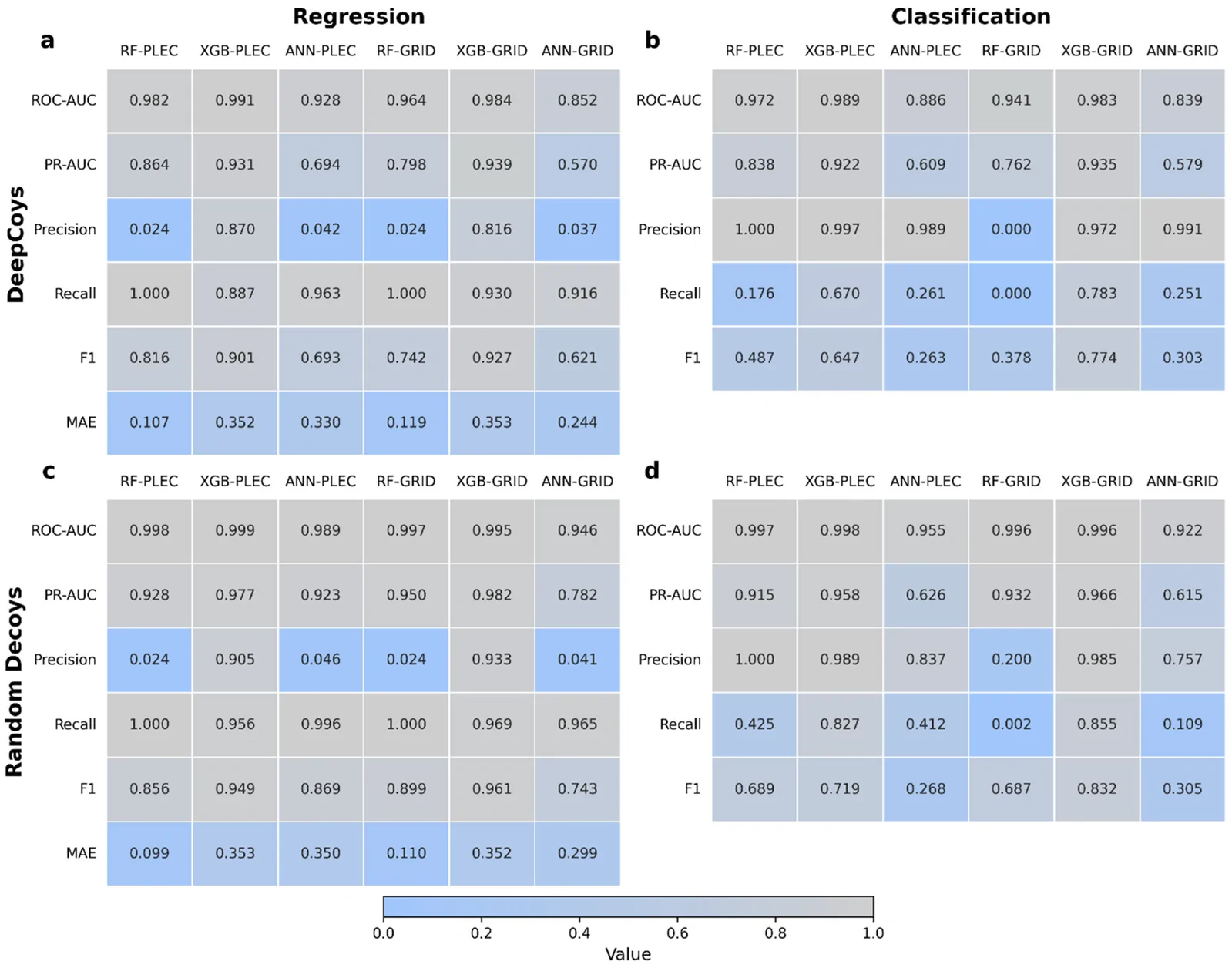
Heat map illustrating the performance of ML regressors and classifiers during 5-fold CV, in terms of ROC-AUC, PR-AUC, Recall, Precision, F1 and MAE in four scenarios: (**a**) training set with DeepCoy decoys and ML regressor variants, (**b**) training set with DeepCoy decoys and ML classifier variants, (**c**) training set with Random decoys and ML regressor variants, and (**d**) training set with Random decoys and ML classifier variants.

Before the inference stage, cross-validation of ML models is crucial. Sometimes, ML models are overfitted by learning essential features as well as noise-specific details in the dataset. This noise and specifics may not generalize to unseen data. Cross-validation ensures that the ML models are trained and evaluated on different subsets of the training data and helps determine whether the model will generalize well to unseen data (Figure 2). Also, keep in mind the stochastic nature of most ML models; a 5-fold CV was performed and the average value for each metric was calculated. All the models are evaluated using ROC-AUC, PR-AUC, Precision, Recall, MCC, and MAE.

In the classification, regardless of feature choice, the PR-AUC values range from 0.839 to 0.989 when trained with DeepCoys and from 0.928 to 0.998 when trained with Random decoys, with the latter showing higher PR-AUC values due to the simpler nature of Random decoys. XGB models, especially XGB-PLEC and XGB-GRID, achieved higher PR-AUC values of 0.989 and 0.983, respectively, when trained with DeepCoys, showing robust performance and good generalization to unseen data. XGB models continue to show strong performance when trained with Random decoys.

In the regression task, regardless of feature choice, PR-AUC values range from 0.862 (ANN-GRID) to 0.991 (XGB-PLEC) when trained with DeepCoys. The XGB models, particularly XGB-PLEC (0.991) and XGB-GRID (0.984) show high PR-AUC values, indicating strong generalization capabilities to unseen data. XGB also outperforms other ML models when trained with Random decoys. Regardless of ML model variants and feature choice, RF and ANN perform moderately during the 5-fold CV. The key observation from 5-fold CV is that across both regression and classification, XGB models consistently perform well, with higher PR-AUC values, when trained on Random decoys, followed by training with DeepCoys. In case of Precision and Recall, regardless of decoy choice, regressions variants are good at Recall while classification variants are good at Precision (Figure 2).

Overall, our ML models gave stable performance across metrics during the 5-fold CV and showed that there was no major bias in the way our initial data were split into the training and test partitions. Otherwise, our models would have performed much worse during 5-fold CV than on the test set.

### 3.3. Developing MLSFs for SBVS

**Figure 3:**
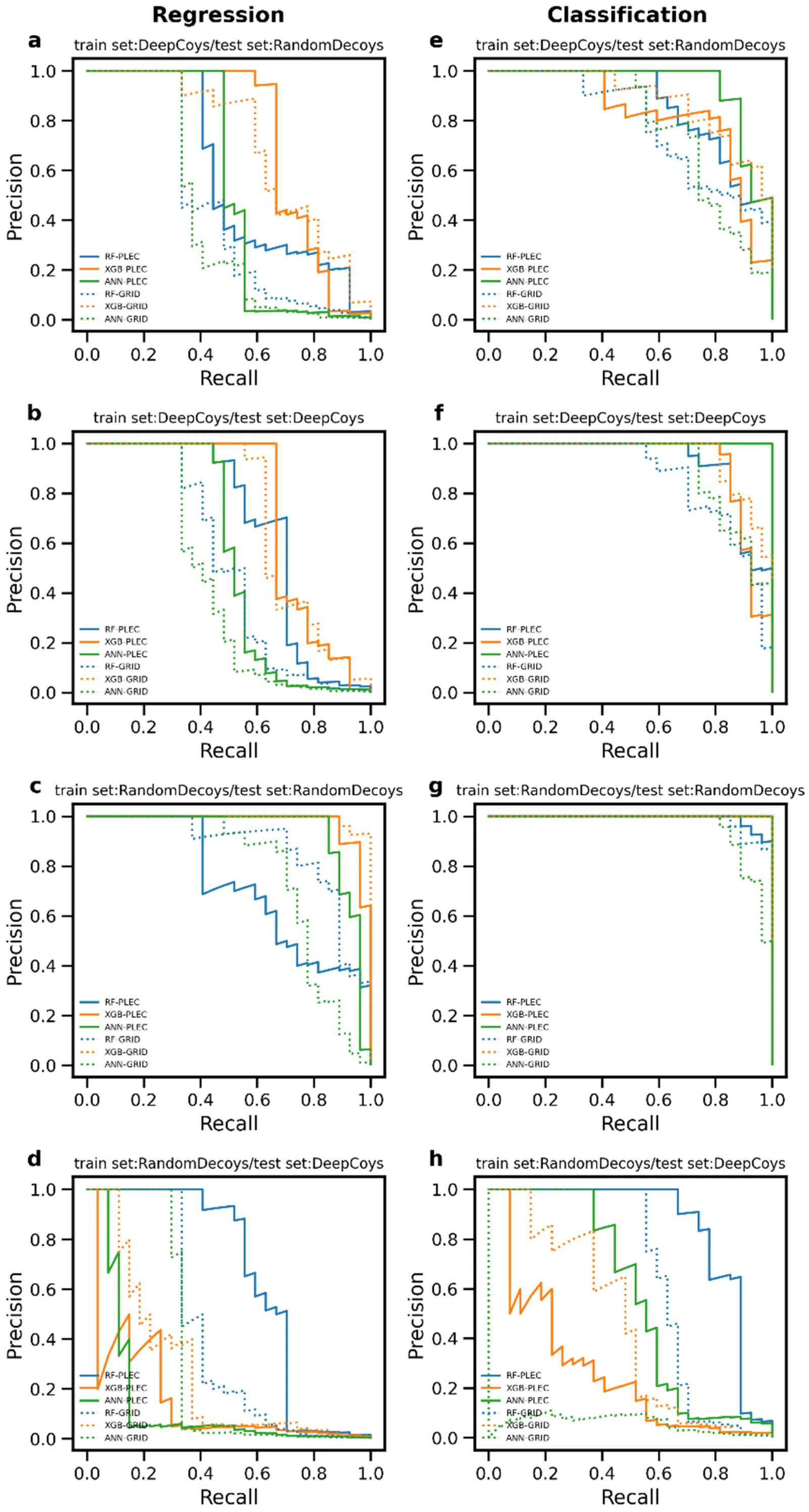
Precision–Recall curves given by TMK-specific SFs. To generate TMK-specific ML SFs, docked poses of the TMK-ligand complex were encoded either as PLEC fingerprints (solid lines) or as GRID features (dotted lines). The resulting features were used by each of the following classification (**right**) and regression (**left**) learning algorithms: RF (Blue), XGB (Orange), and ANN (Green). The PR curve of each target-specific ML SF is that of the training-test run giving a maximum PR-AUC across 10 runs (chosen at random if multiple runs satisfy this criterion). (**a**) training set: DeepCoys/test set: Random Decoys (**b**) training set: DeepCoys/test set: DeepCoys (**c**) training set: Random Decoys/test set: Random Decoys (**d**) training set: Random Decoys/test set: DeepCoys (**e**) training set: DeepCoys/test set: Random Decoys (**f**) training set: DeepCoys/test set: DeepCoys (**g**) training set: Random Decoys/test set: Random Decoys (**h**) training set: Random Decoys/test set: DeepCoys.

Three ML models were trained using two feature types: PLEC and GRID. Both the regression and classification variants of each algorithm were trained. Their performance was evaluated on PR-AUC. The task was to classify molecules as active or inactive using the probability scores of the models. As expected, the models trained on DeepCoy decoys demonstrated high predictive accuracy compared to models trained on Random decoys (Figure 3).

Both training datasets have the same active molecules. DeepCoy decoys closely resemble the active molecules, making it challenging for ML models to distinguish them from active molecules and thus forcing the ML models to learn more realistic and meaningful patterns, which contributes to their overall strong performance. This was also explained by previous studies in which the model trained with the enriched DeepCoy decoys dataset outperformed other MLSFs [22]. Conversely, the model trained with Random decoys struggled to discriminate between active and inactive molecules (Figure 3d,h). This is due to the simplistic nature of Random decoys, which prevents the ML model from learning the realistic features needed to effectively distinguish active and inactive molecules.

The assumption that decoys of the same nature should not be used is supported by the results presented here. Regardless of the ML model’s nature, the models trained and tested with the same nature of decoys, Random decoys, make the ML model overlook the classification task and demonstrate high predictive accuracy (Figure 3f,g).

The high predictive performance demonstrated by these models is not primarily due to the learning capabilities of ML models. Instead, it results from a high bias in both training and test datasets caused by the use of decoys of the same nature. This bias prevents the model from generalizing to real-world scenarios. When these models were tested on test sets having challenging DeepCoy decoys, significantly poorer performance was seen in the case of models trained with Random decoys except RF (Figure 3d,h). This evidence suggests that employing decoys of the same nature in both training and testing phases can be deceptive and should be avoided.

It was also shown that most of the time, PLEC features performed slightly better compared to the GRID features (Figure 3; solid line vs. dotted line). However, statistical analysis showed no statistically significant difference between PLEC and GRID performance (two-sided independent two-sample Student’s *t*-test; *p* = 0.538; see Statistical analysis).

### 3.4. Statistical Analysis of Decoy-Choice Effects on Machine Learning Models for Virtual Screening

**Figure 4:**
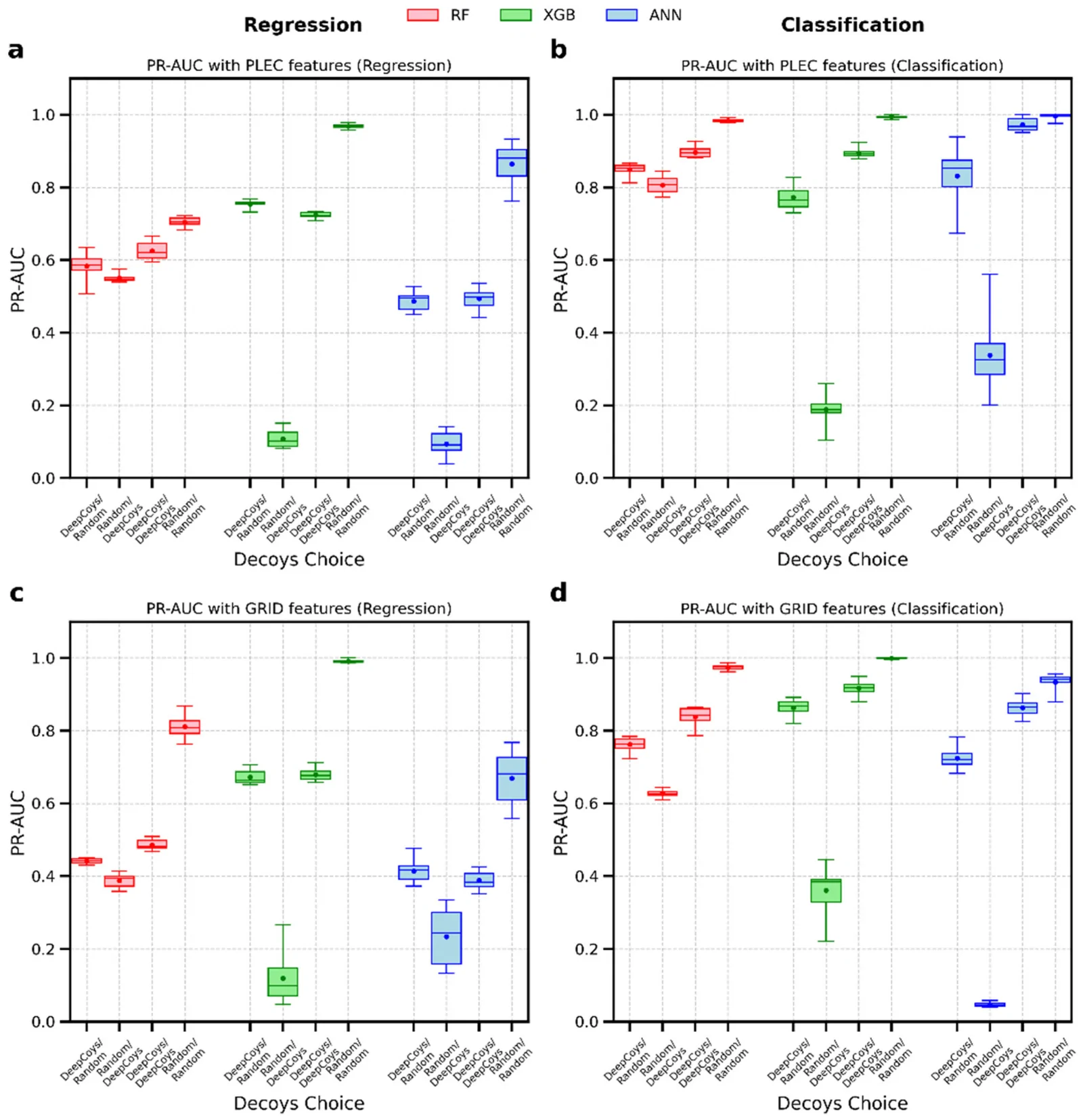
Boxplots illustrating the distributions of PR-AUC values according to several computational modeling choices. These include the nature of decoys in the training and test sets. All training sets and test sets utilize the same active molecules. (**a**) Regression variant with PLEC fingerprints, (**b**) classifier variant with PLEC fingerprints, (**c**) regression variant with GRID features, (**d**) classifier variant with GRID features. For each boxplot, the median value is represented by a horizontal line inside the box and the mean value is represented by a dot.

Figure 4 illustrates the statistical impact of decoy choice on model performance, aligning with the findings discussed earlier. Models trained and tested with the same decoy type perform better compared to those evaluated on cross-decoy scenarios. Specifically, models trained and tested with Random decoys consistently outperform those trained and tested with DeepCoy decoys (last two boxplots in each subfigure of Figure 4). This is likely due to the simpler nature and distinct physicochemical properties of Random decoys compared to active molecules.

On the other hand, models trained with DeepCoy decoys and tested with Random decoys perform better than models trained with Random decoys and tested with DeepCoy decoys (first two boxplots in each subfigure of Figure 4). The poor performance of the latter models is likely due to the simpler nature and distinct physicochemical properties of Random decoys, which prevent the model from learning the more specific features of actives and decoys. All models, regardless of their variants and features, follow the same pattern, except for RF, which performs slightly better when trained with Random decoys and tested with DeepCoy decoys.

To choose the best model, one should focus on models trained with either DeepCoy or Random decoys that perform well on both test sets containing DeepCoy and Random decoys. It is evident from the first and third boxplots in each subfigure that models trained with DeepCoy decoys and tested with Random decoys (first boxplot) and DeepCoy decoys (third boxplot) show minimal differences in performance, owing to their overall good performance on both test sets. In contrast, models trained with Random decoys and tested with DeepCoy decoys (third boxplot) and Random decoys (fourth boxplot) show significant differences in PR-AUC values. This indicates that training with Random decoys and testing with DeepCoy decoys results in poor performance due to the challenging nature of DeepCoy decoys.

### 3.5. Evaluating the vs. Performance

**Figure 5:**
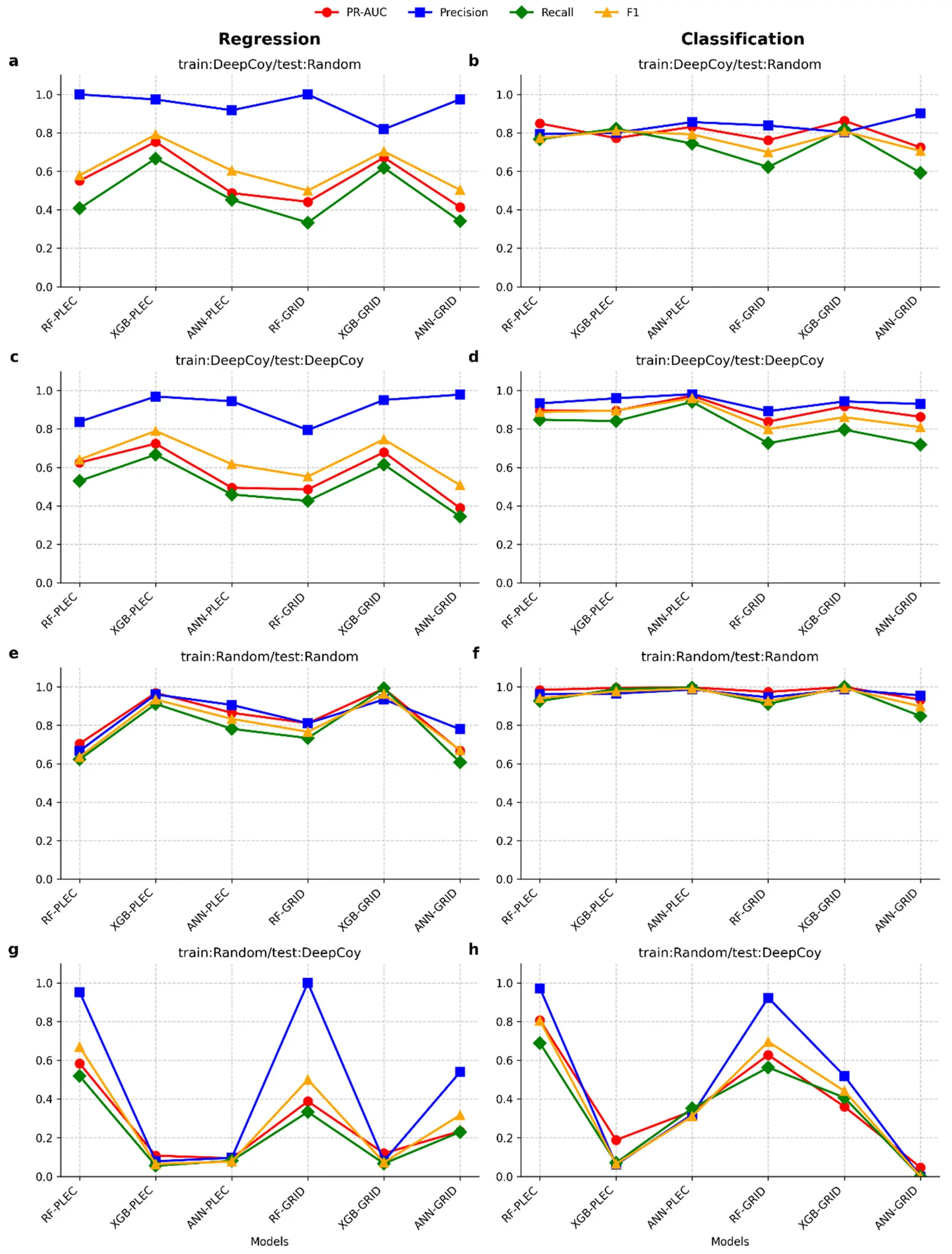
Evaluating the performance of ML regressors and classifiers on test sets during ten train-test runs, in terms of PR-AUC, Recall, and Precision; left column: regressor variant; right column: classifier variant. Each value is the median value of the ten values for each metric. (**a**) training set: DeepCoys/test set: Random Decoys (**b**) training set: DeepCoys/test set: Random Decoys (**c**) training set: DeepCoys/test set: DeepCoys (**d**) training set: DeepCoys/test set: DeepCoys (**e**) training set: Random Decoys/test set: Random Decoys (**f**) training set: Random Decoys/test set: Random Decoys (**g**) training set: Random Decoys/test set: DeepCoys (**h**) training set: Random Decoys/test set: DeepCoys.

The virtual screening performance was evaluated in terms of PR-AUC, F1-Score, Precision, and Recall (Figure 5). Precision focuses on false positives, as it demonstrates how many actual active molecules are among the predicted active molecules. High Precision reflects a lower number of false positives. In the same way, Recall focuses on false negatives, as it demonstrates that many active molecules were predicted as negatives. High Recall means a lower number of false negatives. F1 is the harmonic mean of Precision and Recall.

These metrics are important in virtual screening performance. Each value of the metric in Figure 5 is the average of ten experiments. We restricted our evaluations to two test sets: one containing Random decoys (easier to identify) and one containing challenging decoys. The classifier variant will be discussed as it gave high PR-AUC values compared to the regression variant.

Figure 5 clearly demonstrates that models trained using DeepCoy decoys perform well on both test sets (Figure 5a–d), with the classification variant showing greater predictive power (Figure 5b,d). In contrast, models trained with Random decoys perform well when tested on a dataset containing Random decoys (Figure 5e,f) but perform poorly when tested on a dataset containing DeepCoy decoys (Figure 5g,h).

**Figure 6:**
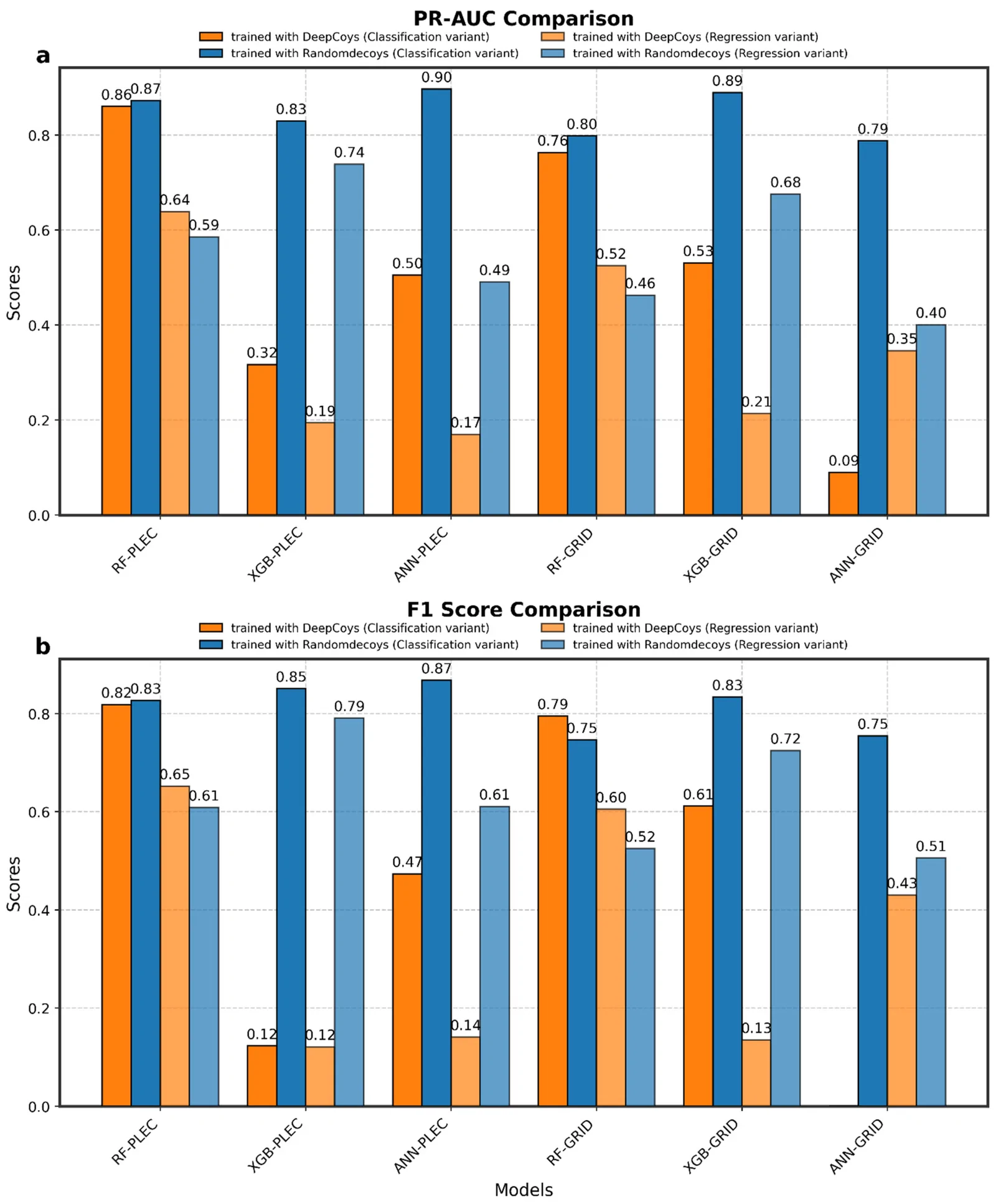
Comparison of model performance using mean PR-AUC and mean F1 Score metrics. (**a**) Each bar shows the harmonic mean of the PR-AUC from two test sets. Orange bars represent the model trained with DeepCoy decoys (classification variant of each model), and blue bars represent the models trained with Random decoys. Light orange and light blue indicate the regression variants. (**b**) Each bar shows the harmonic mean of the F1 score from two test sets. The results highlight the impact of decoy type on model performance, with DeepCoy decoys generally yielding better evaluation scores compared to Random decoys.

To determine the best model, the harmonic mean of the PR-AUC and F1 scores from two test datasets was calculated for models trained using DeepCoy decoys and Random decoys (Figure 6). The harmonic mean was chosen because it emphasizes the balance between the two test sets, penalizing significant disparities. This approach ensures that a model performs consistently well across both datasets, rather than excelling on one while performing poorly on the other. The models trained on DeepCoy decoys generally outperform those trained on Random decoys across all model types (RF, XGB, and ANN) (Figure 6).

For instance, the ANN-PLEC model trained on DeepCoy decoys achieved the highest PR-AUC of 0.90, a notable improvement over the Random decoys’ PR-AUC of 0.50 (Figure 6a). The regression variants perform poorly compared to the classification variant. Similarly, the XGB-PLEC model trained on DeepCoy decoys scored 0.83, compared to a significantly lower score of 0.32 for the Random decoys. This trend is consistent across the remaining models, where DeepCoy decoys result in higher PR-AUC values, indicating better model performance in identifying true positives relative to false positives. The F1 score analysis follows a similar pattern (Figure 6b).

The ANN-PLEC model again leads with an F1 score of 0.87 when trained on DeepCoy decoys, which is substantially higher than the 0.47 achieved when trained on Random decoys. The XGB-PLEC model also shows a significant improvement, with the DeepCoy-trained model achieving an F1 score of 0.85, compared to only 0.12 for the Random decoys. These results suggest that models trained on DeepCoy decoys not only achieve better PR-AUC but also demonstrate superior Precision and Recall, leading to more balanced and reliable classification performance. Based on the combined evaluation of PR-AUC, F1 score, and their harmonic mean, the ANN-PLEC model trained with DeepCoy decoys emerges as the best model. This model consistently outperforms all others across both test sets, demonstrating superior ability to classify active compounds accurately while maintaining a balance between Precision and Recall. Therefore, it is the most suitable model for the classification task.

### 3.6. Analysis of Regression Models for Predicting Binding Affinity

**Figure 7:**
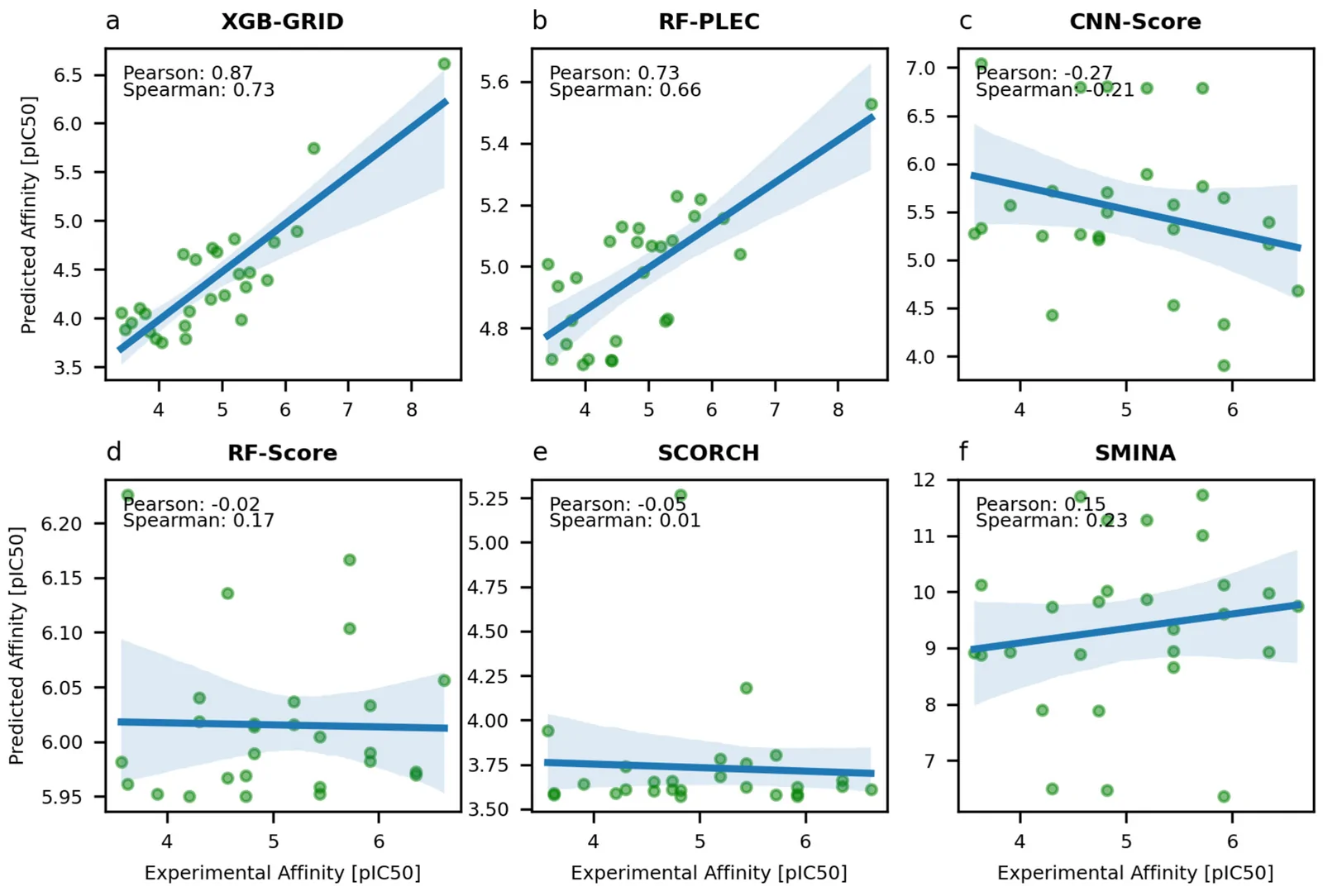
Comparison of predicted vs. experimental binding affinities (pIC50) using different scoring methods. XGB-GRID: Shows a strong positive correlation (Pearson: 0.87, Spearman: 0.73) between predicted and experimental affinities.

In our study, we have evaluated the binding affinity prediction capabilities of regression variants using two statistical analyses: Spearman correlation and Pearson correlation. The statistical analysis indicated that all the models trained with DeepCoy or Random decoys exhibited poor performance in the binding affinity prediction task. The poor performance may be attributed to assigning a pIC50 value of 2 to all decoys. RF and ANN showed negative correlations when trained with decoys, regardless of feature choice, while XGB demonstrated moderate performance with decoys. The performance improved when all models were trained with actives only. Among all models, XGB-GRID emerged as the most predictive model (Figure 7). The Pearson correlation coefficient for XGB-GRID was 0.87, indicating a strong linear relationship between predicted and experimental binding affinities, meaning that the predicted affinities increase linearly with experimental affinities. The RF-PLEC had a Pearson correlation coefficient of 0.73.

The Spearman correlation coefficient, which shows the rank-order correlation between predicted and experimental values for XGB-GRID, was 0.73, while for RF-PLEC was 0.66. This implies that XGB-GRID ranks the molecules based on predicted binding affinity similarly to the experimental ranking. When RF is trained with GRID features, the Pearson and Spearman correlation coefficients were 0.71 and 0.68, respectively. These analyses demonstrated that GRID features are more predictive than PLEC features in binding affinity predictions. In summary, XGB-GRID emerged as a favorable SF for the binding affinity task, followed by RF-PLEC.

### 3.7. Early Enrichment Analysis of TMK-Specific SF

In the virtual screening task, EF1% and NEF1% are the most important metrics for evaluating the effectiveness of SFs. EF1% measures the ratio of active compounds in the top 1% of the ranked list compared to the overall percentage of active compounds. This is crucial for identifying a highly enriched set of active compounds early in the screening process, which can significantly accelerate drug discovery by focusing resources on the most promising candidates [23,49]. The EF1% value depends on the number of active and inactive molecules in the dataset, so NEF1% is the most favorable metric instead of EF1%. NEF1% addresses the variability in the best possible EF1% values. NEF1% facilitates the comparison of machine learning models with classical SFs in terms of performance, across different targets and datasets. Normalization is very beneficial when working with varied datasets, since it guarantees that the performance measures are not influenced by the specific attributes of any individual dataset.

**Figure 8:**
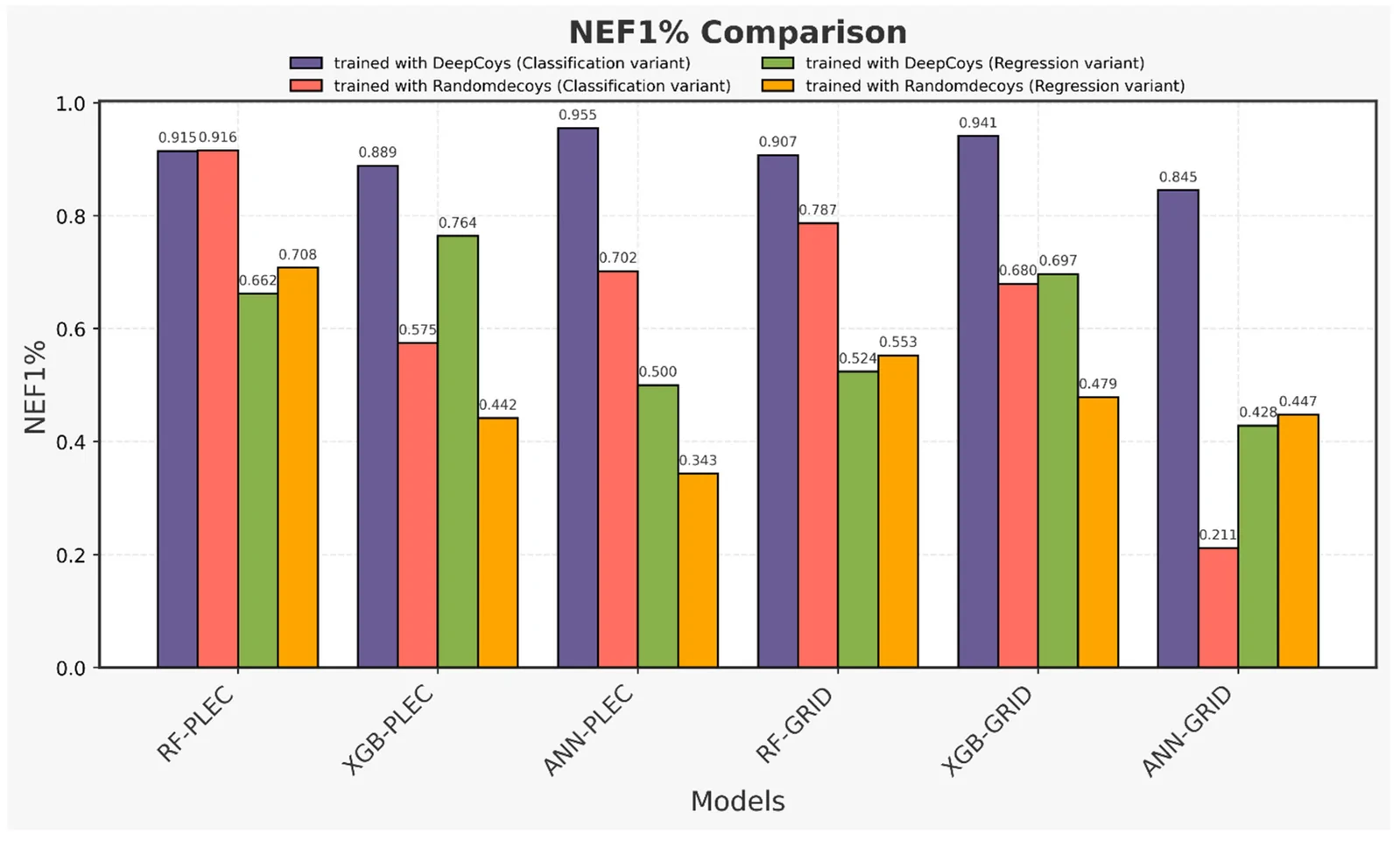
The boxplots illustrating the distributions of NEF1% values for classification models. Each bar shows the harmonic mean of the NEF1% from two test sets. Blue bars represent the model trained with DeepCoy decoys and orange bars represent the model trained with Random decoys.

Across all models, the NEF1% values with DeepCoy decoys are consistently higher compared to Random decoys (Figure 8). This finding underscores the ability of the models to identify actives in the presence of more challenging, structurally similar decoys. Models trained with GRID features, particularly RF-GRID and XGB-GRID, exhibit the highest NEF1% values (0.94 and 0.91, respectively), showcasing their superior enrichment ability under complex conditions. The ANN-PLEC model achieves the best performance overall (NEF1% = 0.96), which highlights the strength of the PLEC fingerprint when paired with neural network architectures.

### 3.8. Evaluation of Generic SF

**Figure 9:**
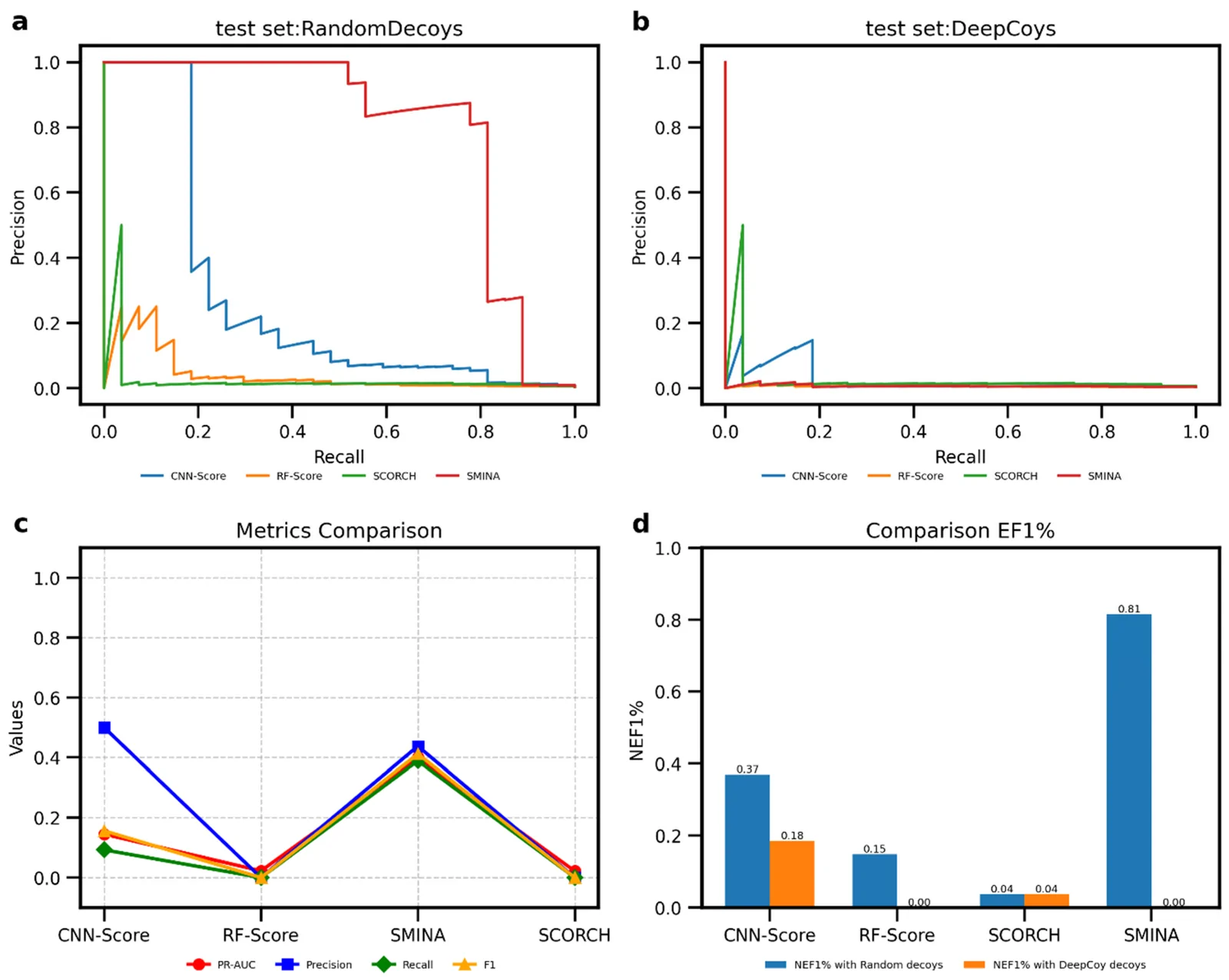
Performance evaluation of generic SFs for CNN-Score, RF-Score, SCORCH, and SMINA: Precision-Recall curves for the test set with (**a**) DeepCoy decoys (**b**) Random decoys (**c**) comparison of various metrics (PR-AUC, Precision, Recall, F1). SMINA and CNN-Score consistently perform better in terms of ROC-AUC and PR-AUC. (**d**) EF1% and EF1max: SMINA achieves the highest EF1% and EF1max, while CNN-Score also shows competitive performance.

Generic SFs are designed to predict binding affinity between protein and ligand. These SFs are trained with a wide range of protein–ligand complexes and are applicable to many targets. In this study, we evaluated the classical generic SF Smina and three machine learning SFs, RF-Score [50], CNN-Score [51] and SCORCH [52] (Figure 9). As generic SFs are widely used for their broad applicability, they often lack the specificity that is needed by a particular target to predict their binding affinity with molecules [30]. This limitation arises from the diverse nature of the training data used to develop these SFs, which may not capture the unique characteristics of specific protein–ligand interactions. On the other hand, developing target-specific machine learning SFs (MLSFs) can significantly enhance predictive performance [22,23]. These models are trained on data specific to a particular target, allowing them to learn the intricate details of that target’s binding interactions. This specialization leads to higher accuracy and better discriminative power in distinguishing active compounds from decoys.

Among the generic SFs, Smina managed to identify active molecules and obtained the highest PR-AUC of 0.2687, but it is still far behind our target-specific SFs such as ANN-PLEC, with a PR-AUC value of 0.90. However, with DeepCoy decoys in the test set, the performance is very poor for all generic SFs. Smina also obtained the highest F1 score among generic SFs, while the F1 score for ANN-PLEC is 0.95, showing superior performance over generic SFs.

### 3.9. Modeling Choices

**Figure 10:**
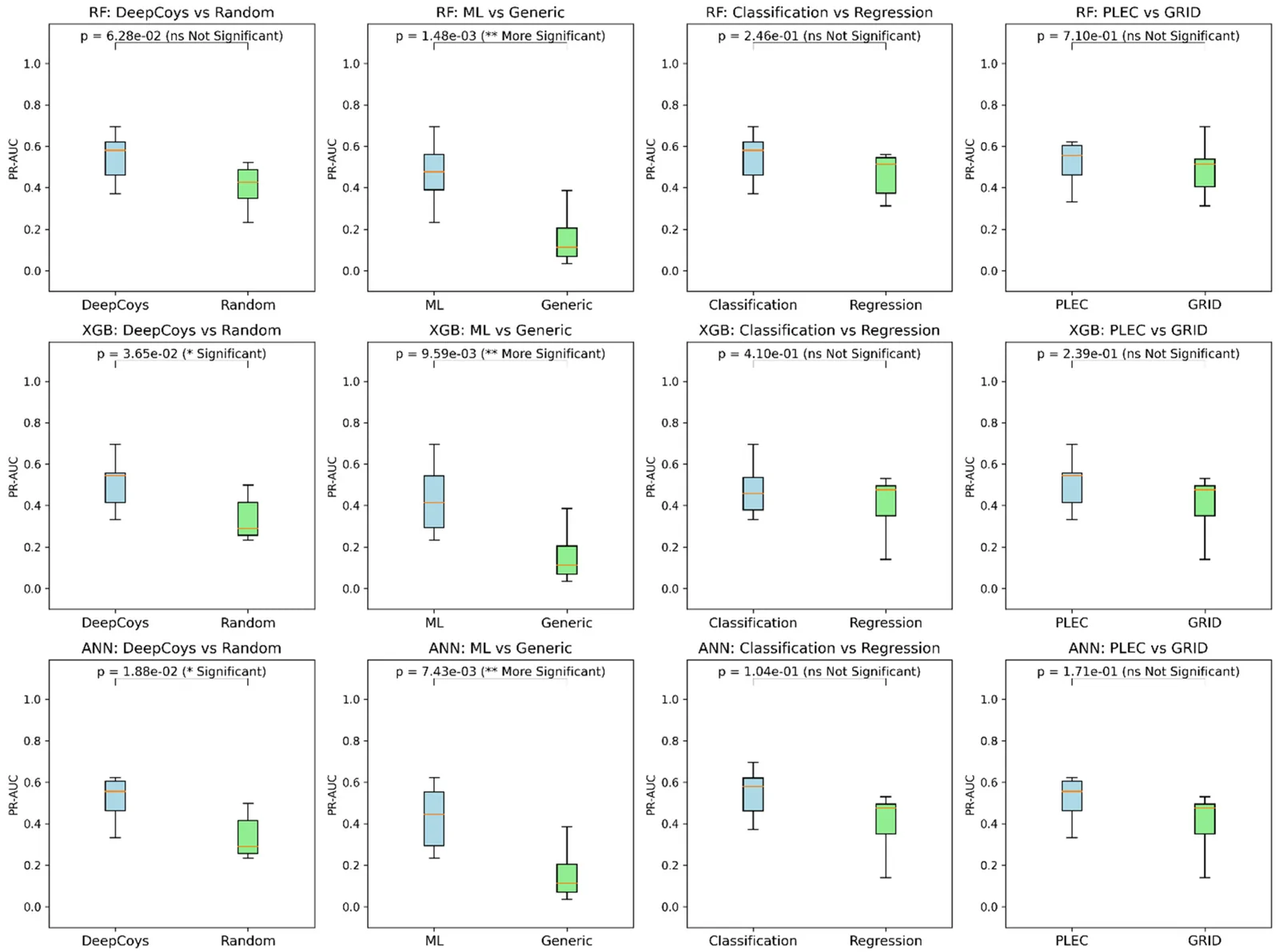
Boxplots comparing the performance of Random Forest (RF), XGBoost (XGB), and Artificial Neural Networks (ANN) models across different experimental settings using Precision–Recall Area Under the Curve (PR-AUC) as the performance metric. The comparisons include (1) DeepCoy vs. Random decoys, (2) ML vs. Generic model types, (3) Classification vs. Regression tasks, and (4) PLEC vs. GRID feature representations. The *p*-values are provided for each comparison, with statistical significance indicated as follows: *p* < 0.01 (** more significant), *p* < 0.05 (* significant), else not significant. Notably, XGB with GRID features showed strong performance in binding affinity prediction, but statistical analysis revealed no significant difference from XGB with PLEC features (*p* = 2.39 × 10^−1^). This analysis emphasizes the influence of decoy choice and model type on performance, while task type and feature representation choices have a lesser impact.

The modeling choices in this study were evaluated through statistical comparisons of different factors affecting model performance, including decoy selection, model type, task type, and feature representation (Figure 10). The Precision–Recall Area Under the Curve (PR-AUC) was used as the primary metric to assess the predictive capability of the models. The choice of decoy selection (DeepCoy vs. Random) had a statistically significant impact on performance for XGBoost (XGB) and Artificial Neural Networks (ANN), with *p*-values of 3.56 × 10^−2^ and 1.88 × 10^−2^, respectively, indicating that DeepCoy decoys improved predictive performance. However, for Random Forest (RF), the difference was not statistically significant (*p* = 6.28 × 10^−2^), suggesting that the influence of decoy selection may vary depending on the model. The comparison between machine learning-based (ML) and generic models revealed a highly significant difference across all models, with ML-based models consistently outperforming generic models. The *p*-values for RF (1.48 × 10^−3^), XGB (9.59 × 10^−3^), and ANN (7.43 × 10^−3^) confirmed the superiority of ML-based models in predicting binding affinity. Conversely, the comparison between classification and regression tasks showed no significant differences in performance across all three models (RF: *p* = 2.46 × 10^−1^, XGB: *p* = 4.10 × 10^−1^, ANN: *p* = 1.04 × 10^−1^), suggesting that the choice between classification and regression does not substantially impact predictive accuracy. Similarly, feature representation (PLEC vs. GRID) did not yield statistically significant differences in performance for any of the models (RF: *p* = 7.10 × 10^−1^, XGB: *p* = 2.39 × 10^−1^, ANN: *p* = 1.71 × 10^−1^). However, despite the lack of statistical significance, XGB with GRID features exhibited strong predictive performance in binding affinity prediction. This suggests that while statistical tests do not confirm a clear advantage of GRID over PLEC, GRID-based feature representation may still be a promising choice for certain applications.

It is important to note that modeling choices are highly target-dependent, as demonstrated in studies such as those by Pedro Ballester, where different targets required different optimal modeling strategies. This highlights the necessity of systematically evaluating various modeling combinations to identify the most effective approach for a given target. A comprehensive assessment of multiple configurations is crucial to optimizing predictive performance and ensuring robust model selection.

### 3.10. Limitations and Future Directions

Several limitations follow from the composition of the datasets used in this study. The decoys that dominate both the training and the test sets, at 50 per active molecule, are computational constructs whose inactivity is assumed rather than measured, and that assumption is not guaranteed to hold for every compound. DeepCoy decoys are by design matched to the actives in physicochemical property space while being topologically distinct from them [32], and property matching necessarily places a proportion of the decoys close to the region of chemical space occupied by genuine TMK-MTB binders. It is therefore plausible that a small fraction of these molecules would exhibit measurable activity against TMK if they were assayed; in other words, they are latent actives mislabeled as negatives. The doppelganger score applied during decoy selection mitigates this risk without eliminating it: by capping the maximum structural similarity between any decoy and the active set, it limits the chance that a decoy is a close analog of a known binder, but it cannot detect an active scaffold that is structurally unrelated to every compound in our active set. Random ZINC20 decoys are less likely to be latent actives in the analog sense, yet ZINC20 is a general-purpose screening library whose members have likewise never been profiled against TMK-MTB.

The practical consequences of this are twofold. Label noise of this kind penalizes the better models disproportionately, because a scoring function that correctly ranks a latent active highly is recorded as having produced a false positive. The Precision, F1, PR-AUC and NEF1% values reported here should therefore be read as conservative lower bounds rather than exact estimates, and this applies most strongly to the DeepCoy-trained models, whose decoys are the most active-like. At the same time, the absolute values should not be interpreted as prospective hit rates, since no amount of cross-validation can correct for a systematically mislabeled negative class. The only definitive remedy is experimental: prospective enzymatic assays on a sample of top-ranked decoys would establish what fraction of the presumed non-binders is genuinely inactive and would allow the reported metrics to be recalibrated accordingly. Enlarging the pool of experimentally confirmed inactives, for example by mining high-throughput screening deposits and patent data as has been demonstrated for PDL1 dimerizers [23], is the most direct route to removing this source of uncertainty, and we regard it as a priority for the next iteration of TMK-VS-Pred.

A second direction for improvement concerns the representation of three-dimensional structure. Molecular recognition is governed by the spatial arrangement of ligand and protein atoms, and both representations employed here capture that geometry only partially. PLEC fingerprints hash the extended-connectivity environments of protein and ligand atoms that fall within a 4.5 Å cutoff and therefore encode which atom pairs are in contact rather than the geometry of the contact; interaction directionality, hydrogen-bond and halogen-bond angles, the relative orientation of aromatic rings and the overall shape complementarity of the pocket are largely discarded by the hashing step. GRID features retain somewhat more spatial information, since SPLIFs are binned by intermolecular distance (0–2 Å, 2–3 Å and 3–4.5 Å) and hydrogen bonds and salt bridges are counted within defined distance windows, but the 16 Å box used here is coarse, and the resulting features remain rotationally invariant summaries rather than an explicit description of the complex. A further simplification is that each complex is represented by a single top-scoring docked pose, so that neither pose uncertainty nor the conformational ensemble accessible to a flexible ligand enters the model input.

Richer three-dimensional inputs are consequently a promising avenue for improving TMK-VS-Pred. Voxelized atomic-density grids processed by three-dimensional convolutional neural networks, as used in CNN-Score [52] and related architectures, learn interaction patterns directly from the occupied volume of the complex instead of from pre-defined descriptors. Graph neural networks built on the protein–ligand interaction graph, and in particular rotation- and translation-equivariant message-passing networks, encode interatomic distances and angles natively and have been reported to outperform fingerprint-based models on affinity-prediction benchmarks. Moving beyond single-pose inputs, representing each ligand by an ensemble of docked poses or of low-energy conformers and aggregating predictions across that ensemble would allow a model to express pose uncertainty rather than inherit the choice made by the docking program. Accounting for receptor flexibility is particularly pertinent for TMK-MTB, whose LID region and P-loop rearrange upon phosphorylation and ligand binding and whose conformational response to a bound inhibitor is invisible to a rigid-receptor representation. Short molecular dynamics simulations of the docked complexes, or interaction-persistence features derived from such simulations, offer a further route to encoding the time-resolved geometry of binding. We anticipate that the decoy-choice effects reported here will persist under these richer representations, since they are a property of the training data rather than of the featurization, and testing that expectation with explicitly three-dimensional models is a natural continuation of the present work.

A further and important limitation is that the present study is entirely retrospective. TMK-VS-Pred has been evaluated on held-out partitions of a curated benchmark, but it has not been deployed in a prospective screen, and no compound ranked by it has been tested experimentally against TMK-MTB. Retrospective enrichment, however carefully the benchmark is constructed, is a proxy for prospective utility rather than a demonstration of it, and the gap between the two is well documented for machine-learning scoring functions. The claims made here are consequently claims about relative model behavior—in particular about the effect of decoy choice on measured and transferable performance—and not claims that TMK-VS-Pred has yielded validated anti-TB hits. A prospective campaign in which the ANN-PLEC classifier and the XGB-GRID regressor are applied to a large commercial library such as ZINC20, in which the top-ranked candidates are examined for their predicted binding modes in the TMK-MTB active site, and in which a shortlist is submitted to enzymatic inhibition assays, is the necessary next step and is planned as the direct continuation of this work. Only such a campaign can establish the true hit rate of the framework.

Relatedly, although the similarity-constrained Butina clustering split described in the Materials and Methods was designed to prevent structural leakage between the training and test partitions, generalization to genuinely novel chemotypes would be probed more directly by a complementary partitioning based on Bemis–Murcko scaffolds, in which every molecule sharing a given ring scaffold is confined to a single partition. Because the two schemes control chemotype overlap by different criteria, a scaffold-split replicate of the full training and evaluation protocol would provide a valuable supplementary assessment of whether the decoy-choice effects reported here persist when the train–test boundary is drawn along scaffold rather than fingerprint-similarity lines. We expect that they would, since the effect originates in the composition of the negative class rather than in the partitioning of the actives, but we regard this as a hypothesis to be tested rather than an established result.

## 4. Conclusions

This study introduces TMK-VS-Pred, a machine learning-based scoring function specifically designed for structure-based virtual screening (SBVS) of thymidylate kinase (TMK) in Mycobacterium tuberculosis. Our findings underscore the critical importance of using challenging decoys, such as DeepCoy decoys, for training machine learning models to enhance generalization and robustness. Models trained on DeepCoy decoys consistently demonstrated superior predictive performance compared to those trained on Random decoys, particularly when tested on challenging datasets. The ANN-PLEC model emerged as the most effective classifier, achieving a PR-AUC of 0.90 and an NEF1% of 0.96 and maintaining strong performance across both the Random and DeepCoy test sets. For binding affinity prediction, XGB-GRID proved to be the most predictive model, with Pearson and Spearman correlation coefficients of 0.87 and 0.73, respectively, indicating its capability to accurately rank molecules by binding affinity.

The results also highlighted that models trained on Random decoys exhibited inflated performance when tested on similar decoys, but their performance degraded significantly on DeepCoy decoys, emphasizing the need for diverse and realistic decoy sets in SBVS tasks. Additionally, our comparison with generic scoring functions (Smina, RF-Score, SCORCH, and CNN-Score) revealed the clear superiority of target-specific machine learning scoring functions in both classification and regression tasks. TMK-VS-Pred achieved significantly higher PR-AUC and NEF1% values, particularly in scenarios involving challenging decoys, highlighting its potential to accelerate the discovery of anti-TB compounds.

Furthermore, our findings demonstrate the impact of feature representation, with PLEC fingerprints performing slightly better for classification tasks, while GRID features showed greater predictive power in binding affinity regression. This dual approach of leveraging multiple feature types ensures comprehensive modeling of protein–ligand interactions. The combination of ANN-PLEC for classification and XGB-GRID for regression tasks offers a versatile framework for drug discovery.

In conclusion, TMK-VS-Pred addresses critical limitations of generic scoring functions and provides a robust, target-specific solution for SBVS. By integrating challenging decoys, advanced feature representations, and tailored machine learning algorithms, this study sets a new benchmark for virtual screening methodologies. Future research can build upon this framework by exploring additional featurization techniques, expanding to other protein targets, and integrating deep learning architectures to further improve predictive performance in drug discovery pipelines. TMK-VS-Pred represents a significant step forward in combating tuberculosis through data-driven, structure-based virtual screening.

Three caveats should accompany these conclusions. First, the negative class in our benchmarks consists predominantly of computationally generated or randomly selected decoys whose inactivity against TMK-MTB is assumed rather than experimentally verified, so that the reported metrics are benchmark estimates that await prospective validation, and a small fraction of the decoys may prove to be measurably active. Second, the PLEC and GRID representations encode protein–ligand geometry only indirectly and from a single docked pose, which leaves explicit three-dimensional and conformational-ensemble representations, such as three-dimensional convolutional and equivariant graph neural networks, as a clear opportunity for further gains.

Third, the evaluation presented here is retrospective: TMK-VS-Pred has not yet been applied prospectively, and no candidate ranked by it has been assayed against TMK-MTB, so the framework’s true hit rate remains to be established. A prospective screen of a commercial library with the ANN-PLEC classifier and the XGB-GRID regressor, followed by enzymatic validation of the top-ranked candidates, is the direct next step of this work.

## Figures and Tables

**Table 1:** Training and test partitions.

Partition ID	Training Set	Test Set	Model Type
A	DeepCoys	Random Decoys	Classification
B	DeepCoys	DeepCoys	Classification
C	RandomDecoys	Random Decoys	Classification
D	RandomDecoys	DeepCoys	Classification
E	DeepCoys	Random Decoys	Regression
F	DeepCoys	DeepCoys	Regression
G	RandomDecoys	Random Decoys	Regression
H	RandomDecoys	DeepCoys	Regression

## Data Availability

The data used in this study and all codes to reproduce this study are freely available at https://github.com/JuniML/TMK-VS-Pred (accessed on 2 January 2026). The DeepCoy decoys are generated through the deepcoy algorithm, and the code is available at https://github.com/fimrie/DeepCoy/tree/master (accessed on 2 January 2026).
